# Cell lines from circulating tumor cells

**DOI:** 10.18632/oncoscience.195

**Published:** 2015-08-20

**Authors:** Klaus Pantel, Catherine Alix-Panabières

**Affiliations:** Laboratory of Rare Human Circulating Cells (LCCRH), Department of Cellular and Tissular Biopathology of Tumors, University Medical Centre, Montpellier, France

**Keywords:** circulating tumor cells (CTC), CTC line, solid cancer

Circulating tumor cells (CTCs) in the blood of cancer patients have received increasing attention as new diagnostic tool enabling “liquid biopsies” [1]. The perspective to avoid invasive tissue biopsies in the future and obtain similar or even more information by a “simple” blood test has enormous implications in cancer diagnostics [2]. In contrast to the wealth of descriptive studies demonstrating the clinical relevance of CTCs as biomarker, the extremely low concentration of CTCs in the peripheral blood of most cancer patients challenges further functional studies. A prerequisite for functional analyses was therefore the recent advances in our ability to culture CTCs *in vitro* or expand the CTC pool *in vivo* using xenografts.

The recent manuscript by Cayrefourcq *et al.* focused on how to establish a colon CTC line and its key characteristics have been reported [3]. In this study, they applied a negative selection for CTC enrichment on blood samples from 71 patients with metastatic colon cancer and cultured all of the CD45^(−)^ remaining cells in non-adherent culture conditions. The authors have provided the experimental proof that CTCs isolated from the blood of a colon cancer patient are able to give rise to a permanent cell line [3]. To our best knowledge, no other group has published the establishment of a permanent cell CTC line or even transient CTC cultures from patients with colon cancer. One reason is that the frequency of CTCs is lower in peripheral blood of colon cancer patients as compared to breast or prostate cancer patients, making it even more difficult to find and grow CTCs in colon cancer. Besides its capacity to expand *ex vivo* for more than 2 years, the CTC-MCC-41 line showed (*i*) epithelial properties with stem-cell like characteristics, (*ii*) an intermediate epithelial/mesenchymal phenotype, (*iii*) a potential to induce quickly *in vitro* angiogenesis, (*iv*) an osteomimetic signature and (*v*) tumorigenic properties in SCID mice. Importantly, this CTC line shares the main features of the original primary tumor and lymph nodes metastasis of the colon cancer patient.

Recent advances in cell culture technologies have opened a new avenue to develop primary cell cultures or permanent cell lines from CTCs. The first publications on CTC cultures dealt with blood from metastatic breast cancer patients. First, Dario Marchetti's group has established primary cultures from CTCs of breast cancer patients with brain metastases [4]. In EpCAM^(−)^ CTCs, a potential signature of brain metastasis comprising “brain metastasis selected markers (BMSMs)” HER2^(+)^/EGFR^(+)^/HPSE^(+)^/Notch1^(+)^ was identified [4]. CTC lines expressing the BMSM signature were highly invasive and able to generate brain and lung metastases in nude mice. Second, Daniel Haber's group has reported on oligoclonal cultures (sustained *in vitro* for > 6 months) of CTCs isolated from six patients with metastatic breast cancer [5]. Third, in prostate cancer, Gao *et al.* used a 3D organoid system and succeeded to develop a long-term culture from the peripheral blood of one patient with castration-resistant metastatic disease and high CTC counts and PSA levels [6]. Fourth, Sunitha Nagrath's group focused on lung cancer and developed a novel *in situ* capture and culture methodology for *ex vivo* expansion of CTCs using a 3D co-culture model [7].

**Figure 1:**
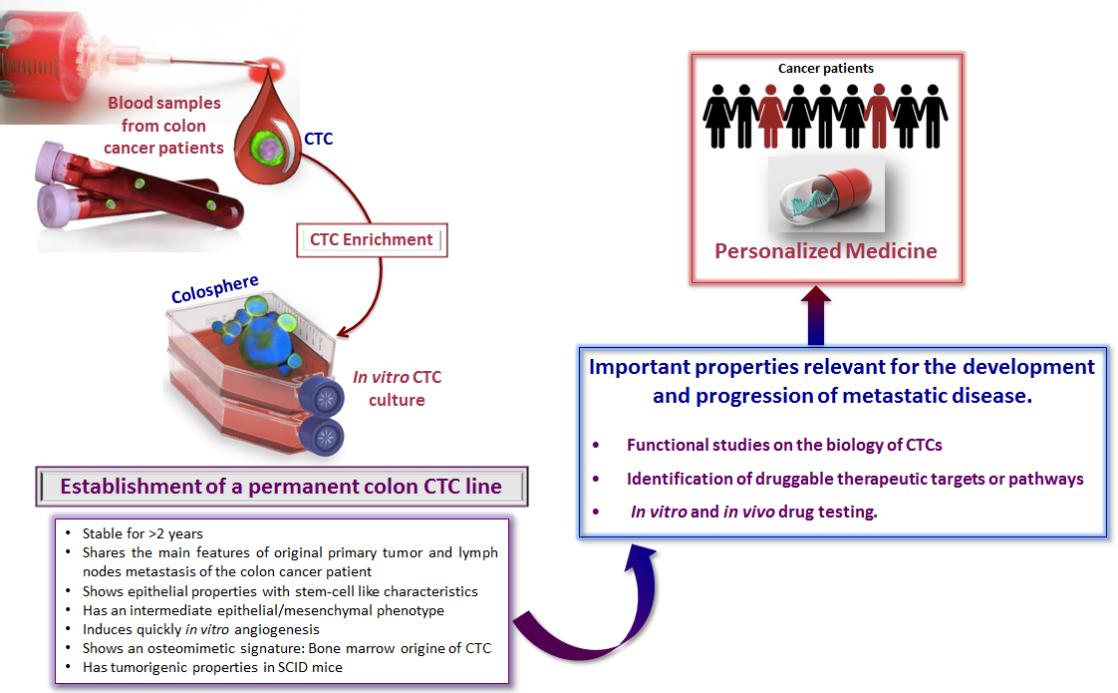
Establishment of a colon CTC line Characteristics and potential applications for basic science and drug development.

CTC lines might be used to test new therapeutic approaches. In this context, novel immunotherapeutic strategies have been developed and antibodies against proteins that block the immune response of T-cells such as PD-L1 or CTLA4 have been approved for treatment of cancer patients. Recently, we could show, for the first time, that PD-L1 is frequently expressed on a variable subset of CTCs obtained from metastatic breast cancer patients [8]. Further functional analysis of this interesting subset might reveal CTC-specific immunosuppressive properties.

Taken together, the establishment of functional CTC cell line models is now feasible. These models might be useful to test new cancer drugs specifically targeting CTCs. However, to achieve this important goal it is crucial to increase the efficiency of establishing CTC cell lines and xenografts and extend the current studies to patients at earlier stages. The low concentration of CTCs in earlier stage patients might be a bottleneck to identify “metastasis-initiator cells” that may be resistant as well to the immune system by expressing PD-L1. Thus, increasing the yield of current CTC assays along with the development of more efficient culture strategies seems to be of utmost importance.

## References

[1] Pantel K, Alix-Panabieres C (2010). Trends Mol Med.

[2] Alix-Panabieres C, Pantel K (2014). Nat Rev Cancer.

[3] Cayrefourcq L (2015). Cancer Res.

[4] Zhang L (2013). Sci Transl Med.

[5] Yu M (2014). Science.

[6] Gao D (2014). Cell.

[7] Zhang Z (2014). Oncotarget.

[8] Mazel M (2015). Mol Oncol.

